# Remote ischemic conditioning for the prevention of stroke-associated pneumonia (RICA-2): protocol for a multicenter, prospective, randomized, double-blind, sham-controlled phase III trial

**DOI:** 10.1186/s13063-025-09140-x

**Published:** 2025-10-16

**Authors:** Lina Jia, Chengbei Hou, Qing Mei, Bowei Zhang, Wenbo Zhao, Heng Zhao, Suhang Shang, Xiuhai Guo, Qingfeng Ma, Haiqing Song, Chuanhui Li, Ran Meng, Weihai Xu, Yilong Wang, Gelin Xu, Chuanjie Wu, Xunming Ji, for the RICA- Trial Investigators

**Affiliations:** 1 Department of Neurology, Xuanwu Hospital, Capital Medical University, Beijing, China; 2 Department of Neurology, Beijing Pinggu Hospital, Beijing, China; 3 Beijing Institute for Brain Disorders, Capital Medical University, Beijing, China; 4 Department of Neurology, Tongren People’s Hospital, Tongren, Guizhou, China; 5 Department of Neurology, The First Affiliated Hospital of Xi’an Jiaotong University, Xi’an, Shaanxi, China; 6 Department of Neurology, Chinese Academy of Medical Sciences & Peking Union Medical College, Beijing, China; 7 Department of Neurology, Beijing Tiantan Hospital, Capital Medical University, Beijing, China; 8 Department of Neurology, First Affiliated Hospital of Shenzhen University, Shenzhen, Guangdong China; 9 Center for Evidence Based Medicine, Xuanwu Hospital, Capital Medical University, Beijing, China

**Keywords:** Remote ischemic conditioning, Acute ischemic stroke, Stroke-associated pneumonia, Clinical trial, Protocol

## Abstract

**Background:**

Stroke-associated pneumonia (SAP) is one of the most common complications in acute ischemic stroke (AIS) patients, affecting 8.5% to nearly 30% of cases and significantly impacting both mortality and long-term survival. As it is closely related to worse outcome, exploring effective preventive strategies, such as remote ischemic conditioning (RIC), is essential for reducing SAP incidence and improving patient outcomes. This study aims to compare the efficacy and safety of RIC for preventing SAP in patients with ischemic stroke within 24 h of symptom onset.

**Methods and design:**

RICA-2 is a multicenter, randomized, double-blind, parallel-controlled, phase III clinical trial in China. This study will enroll an estimated 1650 patients aged ≥ 18 years within 24 h after AIS symptom onset, with National Institutes of Health Stroke Scale ⩾ 4. The patients will be randomly assigned to RIC or Sham-RIC (1:1) and will be treated with cuff inflation at 200 mmHg or 60 mmHg, respectively. This procedure will be administered twice daily for seven consecutive days. The primary efficacy endpoint is SAP incidence rate. Safety incidence will be recorded and reported.

**Discussion:**

RIC has broad clinical application prospects and may play a preventive role in stroke-related pneumonia. RICA-2 is designed to verify whether RIC treatment can serve as an adjuvant therapy for preventing stroke-associated pneumonia and to identify safety concerns.

**Trial registration:**

ClinicalTrials.gov NCT05982015. Registered on January 22, 2024.

**Supplementary Information:**

The online version contains supplementary material available at 10.1186/s13063-025-09140-x.

## Introduction and rationale

Stroke-associated pneumonia (SAP) is one of the most common complications after stroke [1]. SAP is strongly associated with increased mortality rates in stroke patients [2], significantly increasing the risk of death up to 3 years poststroke [3]. During a stroke, local inflammatory activity intensifies while systemic immune function decreases, resulting in immunosuppression, which is a primary independent risk factor for SAP in stroke patients [4]. Given the substantial impact of infections on functional recovery, numerous trials have explored the efficacy of prophylactic antibiotics in stroke care. However, these studies have shown considerable heterogeneity in design, antibiotic type, and measured outcomes, limiting their generalizability [5], as seen in trials such as the Preventive Antibiotics in Stroke Study and STROKE Infection Prevention Study [6, 7]. Therefore, novel treatments aimed at reducing SAP and improving recovery outcomes in stroke patients are urgently needed [8].

Remote ischemic conditioning (RIC) has emerged as a promising nonpharmacological intervention to protect distant organs by inducing periodic ischemia and reperfusion in the limbs. Studies suggest that RIC activates endogenous protective mechanisms, protecting remote organs [9, 10]. In an experimental ischemia–reperfusion model, RIC has been shown to reduce cerebral ischemia–reperfusion injury, inflammation, brain edema, and neuronal apoptosis [11]. Whether RIC can be adopted as an effective treatment to decrease SAP occurrence in patients with AIS remains unclear.

Therefore, this study aimed to evaluate the efficacy and safety of RIC in preventing SAP in patients with ischemic stroke. This multicenter, double-blind, randomized, sham-controlled phase III trial is designed to provide robust evidence on a novel prevention method for SAP, as well as the potential benefits of RIC.

## Methods

### Design

**Fig. 1:**
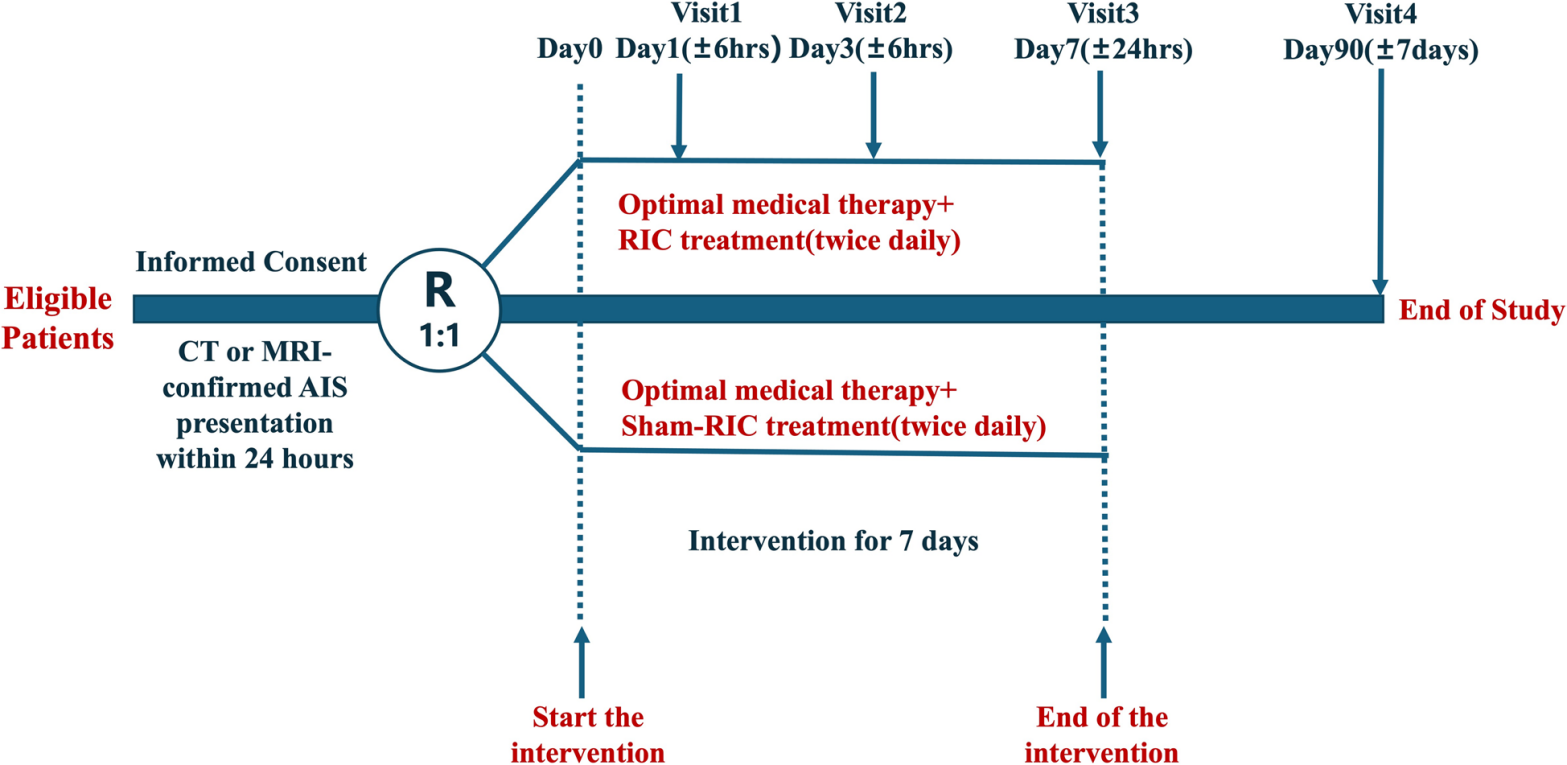
Study design

This study is a multicenter, randomized, double-blind, sham-controlled phase III clinical trial (http://www.clinicaltrials.gov NCT05982015) in China and approved by the Ethics Committee of Xuanwu Hospital of Capital Medical University and other cooperative hospitals. The design is shown in Fig. 1. Patients will be subjected to head computed tomography (CT) or magnetic resonance imaging (MRI) before randomization to identify patients with ischemic stroke. All the subjects received optimal medical therapy on the basis of the 2018 clinical guidelines for AIS [12]. Before each participant is enrolled, the researcher is responsible for providing a comprehensive overview of the study’s purpose, procedures, and potential risks to the patient or his or her representative. The participants or their legally authorized representative signed a written informed consent form and were informed of their right to withdraw from the study at any time.

### Patient population

A total of 1650 patients with acute AIS within 24 h of onset were recruited from approximately 80 medical centers in China according to the inclusion and exclusion criteria in Table 1.

**Table 1:** Patient selection criteria

**Inclusion criteria**
• Age ≥ 18 years old
• Diagnosis of acute ischemic stroke
• RIC or Sham-RIC can be treated within 24 h after stroke onset
• NIHSS score ≥ 4
• Subject or his or her legally authorized representative was able to provide informed consent
**Exclusion criteria**
• During the screening period, body temperature ≥ 38 °C
• Evidence of lung infection, urinary tract infection, or other infectious diseasesduring the screening period
• Expected lifespan less than 7 days
• Mechanical ventilation is expected to be required within 7 days
• Anti-infective drugs were used within 7 days prior to stroke
• Uncontrolled hypertension with medication (defined as systolic blood pressure ≥ 200 mmHg and/or diastolic blood pressure ≥ 110 mmHg)
• There are contraindications for remote ischemic conditioning (such as skin and soft tissue injury, fractures, and peripheral arterial disease in the upper limbs)
• History of autoimmune disease or malignancies
• Use of immunosuppressive drug within the preceding 3 months
• Pregnant or lactating, or pregnancy test positive
• Current participation in another investigational trial
• Other conditions are not suitable for this trial as evaluated by researchers

*RIC* Remote ischemic conditioning. *Sham-RIC *Sham remote ischemic conditioning, *NIHSS* National Institutes of Health Stroke Scale

### Randomization

Randomization will occur after written informed consent is obtained from the patient or his or her guardian. Following successful screening of participants, patients will be randomly assigned to either the RIC group or the Sham-RIC group at a 1:1 ratio via randomized blocks (4, 6, and 8) stratified by age (≤ 70 or > 70 years old), stroke severity (NIHSS scores between 4 and 20 years or higher than 20), and sex (male or female). A central network randomization system will generate random code. Neither patients nor researchers will have information on the group allocation. Both RIC and Sham-RIC will be administered via equipment with the same appearance but with different pressure settings for the two treatment regimens. Additionally, consecutive enrolled subjects will not be placed in the same ward for this study.

At the end of this study, the subjects were asked several questions, as shown in Table 2, to investigate unblinding situations. Unblinding will only be allowed if a participant’s health is at risk and the attending physician deems it necessary. Unblinding must be approved by the principal investigator and documented.

**Table 2:**
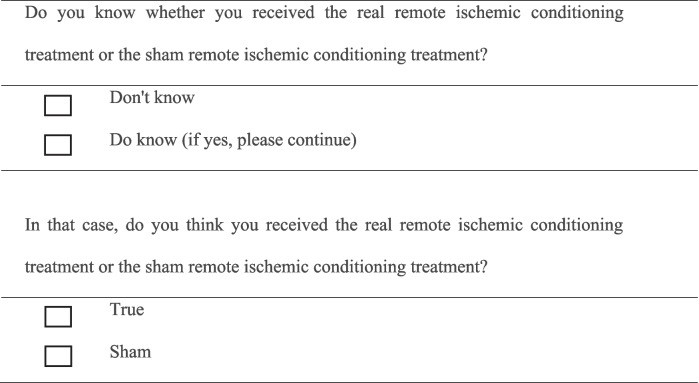
Blinded survey

### Baseline measurements

In addition to confirming eligibility for AIS, a comprehensive baseline assessment will be conducted prior to randomization. This assessment will encompass several key variables, including demographic characteristics, clinical and medical history, physical examination findings, vital signs, smoking and alcohol consumption status, and medication usage within 1 week prior to admission. Additionally, laboratory examinations, neuroimaging, and chest imaging, including CT or chest X-ray scans, will also be performed. Furthermore, the assessment will utilize standardized scales such as the National Institutes of Health Stroke Scale (NIHSS), the modified Rankin scale (mRS), the self-rating depression scale (SDS), the generalized anxiety disorder scale (GAD-7), and a swallowing function assessment.

### Intervention and assessment

Eligible patients will be randomly assigned to either the RIC group or the Sham-RIC group. The RIC procedure consisted of 5 cycles of inflation to a pressure of 200 mmHg (RIC group) or 60 mmHg (Sham-RIC group) and deflation for 5 min alternately within 1 h of enrollment performed on the upper arm by an electric autocontrolled device with cuff (patent number ZL201420846209.5, China); the total process lasted 45 min. The Sham-RIC did not result in upper limb ischemia. All patients will receive optimal medical treatment and care from local medical practices throughout the study. The numbers of RIC treatment will be recorded by the study nurse and signed their names per time. The electronic data collection system will send message to the researcher to remind them to visit patients.

Adherence is defined as completion of at least 10 out of the 14 scheduled RIC sessions (≥ 70%). Participants completing fewer than 10 sessions will be considered non-adherent. In such cases, study staff will promptly contact the participant to determine the reason for non-adherence and provide necessary support. When necessary, additional instruction or clarification will be offered to reinforce the importance of RIC and ensure correct implementation. Regular follow-up communication will be maintained to encourage continued participation and improve adherence.

The research period spans approximately 2 years. Research protocol version 3.0, version date July 19, 2023, recruitment date January 22, 2024, recruitment completed on September 12, 2024. The final follow-up period for the subject is expected to be completed on December 12 (± 7 days), 2024. Follow-up visits will be scheduled at each research center on days 1, 3, 7, and 90 after the initiation of treatment, as well as at the conclusion of the study. The details are outlined in Table 3.

**Table 3:**
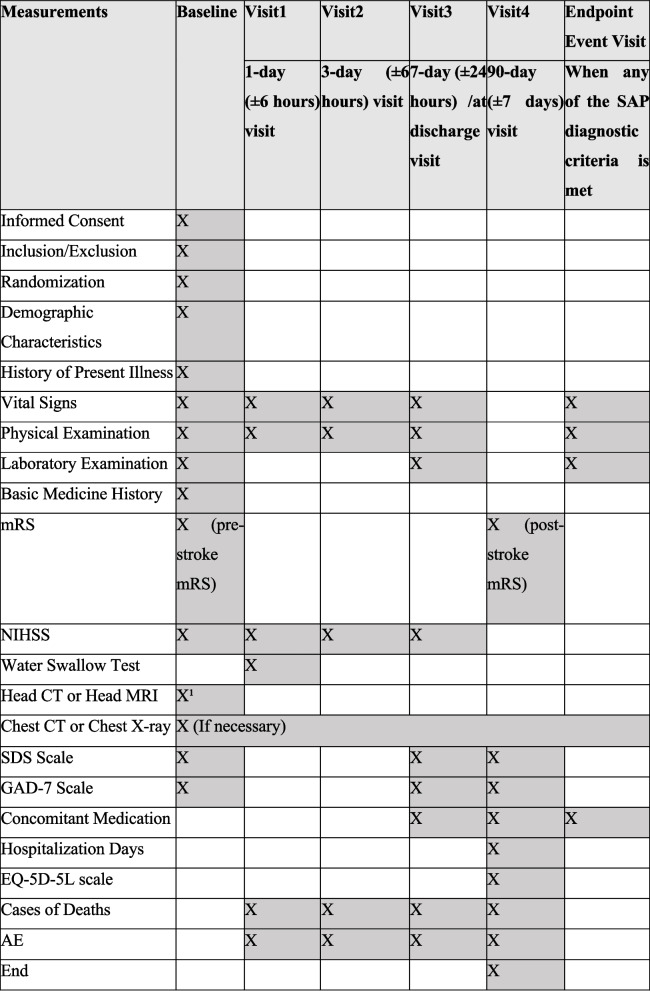
Schedule of activities and assessments

^1^For all enrolled cases, head CT or head MRI examination will be the first choice before randomization. Vital signs are related to blood pressure, heart rate, body temperature, and breathing rate. Physical examination includes weight, height, and limb examination; laboratory examination: white blood cell, red blood cell, platelet, hemoglobin, neutrophil, monocyte, and lymphocyte counts and percentages, C-reactive protein, high-sensitivity C-reactive protein, alanine aminotransferase, aspartate aminotransferase, creatinine, glucose, total cholesterol, triglycerides, high-density lipoprotein, low-density lipoprotein, homocysteine, lipoprotein(a), D-dimer, fibrinogen, and uric acid, *SAP* Stroke-associated pneumonia, *AE* Adverse event, *CT* Computed tomography, *EQ-5D-5L* EuroQoL 5-Dimensions 5-Level questionnaire, *MRI* Magnetic resonance imaging, *mRS* modified Rankin scale, *NIHSS* National Institutes of Health Stroke Scale; *SAE*, serious adverse event

### Outcomes

The primary efficacy endpoint is the incidence of SAP within 7 days of intervention after randomization. The details of the SAP diagnostic criteria [13] are presented in Table 4. The secondary endpoints were as follows: diagnosis of pneumonia by clinical doctors within 7 days; diagnosis of pneumonia by clinical doctors between 8 and 90 days; mRS score of 0–1 ratio on day 90; mRS score of 0–2 ratio on day 90; distribution of mRS scores on day 90; EuroQoL 5-Dimensions 5-Level questionnaire (EQ-5D-5L) score on day 90; NIHSS scores at 24 h after onset; NIHSS scores at day 7; incidence of urinary tract infection within 7 days; incidence of all infections within 7 days; all-cause mortality within 90 days; and total inpatient days. The incidence of adverse events was recorded and reported.

**Table 4:** Suggested diagnostic criteria for definite and probable SAP in non-ventilated patients, based on CDC guidelines

At least one of the following criteria:
1. Fever (beyond 38 °C) with no other recognized cause
2. Leukopenia (lower than 4000 WBC/mm^3^) or leukocytosis (beyond 12,000 WBC/mm^3^)
3. For adults aged beyond 70 years, altered mental status with no other recognized causeAnd at least two of the following symptoms need to be observed:New onset of purulent sputum, a change in sputum character over 24 h, or increased respiratory secretions, or increased suctioning requirementsNew onset or worsening cough, dyspnea, or tachypnea (respiratory rate > 25/min) rales, crackles, or bronchial breath soundsWorsening gas exchange (e.g., O_2_ desaturation such as PaO_2_/FiO_2_ lower than 240, or increased oxygen requirements)And at least two serial chest radiographs must show at least one of the following:New or progressive and persistent infiltrate, consolidation, or cavitationOne definitive chest radiograph is acceptable in patients without underlying pulmonary or cardiac disease

Legend: Definite SAP: all CDC criteria are fulfilled, including diagnostic changes on at least one chest x-ray. CDC refers to the Centers for Disease Control and Prevention; *FiO*_*2*_ Fraction of inspired oxygen, *PaO*_*2*_ Partial pressure of oxygen, *SAP* Stroke-associated pneumonia, and *WBC* White blood cell count

### Sample size

On the basis of previous studies [14] and a recent survey from Xuanwu Hospital, the estimated incidence of SAP in patients with AIS within 24 h of onset is 17%. The sample size calculation assumes that RIC will reduce the SAP rate from 17.0% to 11.9%. With a sample size of 1486 across both groups, the study will have 80% power to detect a difference in SAP incidence between the Sham-RIC and RIC groups at a two-sided significance level of 0.05. While we anticipate that all (or at least a very high proportion) of the enrolled patients will be evaluable at the end of the study, we intend to recruit 875 patients per treatment group, totaling 1650 patients for this study.

### Statistical analysis

The analysis of efficacy endpoints will be conducted on an intention-to-treat basis. The primary endpoint was the incidence of SAP within 7 days postrandomization. To assess the difference in SAP incidence between the two groups (RIC group vs. Sham-RIC group), we used a modified Poisson regression model to analyze the primary efficacy endpoint. Adjustments for stratification factors, including age, sex, and NIHSS score, were incorporated into the model to control for potential confounding effects.

For secondary endpoints, continuous variables will be analyzed via linear regression models, with the same adjustments applied for stratification factors. Binary variables are analyzed via modified Poisson regression, whereas ordinal variables are analyzed via ordinal regression models. Time-to-event variables will be evaluated via Cox proportional hazards regression, with stratification factors controlled throughout.

Missing data for the primary endpoint will be addressed using multiple imputation methods to minimize potential bias. For secondary endpoints, no imputation will be applied. Given the exploratory nature of these outcomes, analyses will be limited to participants with complete data only.

Adverse events will be documented and reported. Statistical significance will be defined as a *P* value < 0.05. This study did not set an interim analysis.

### Study organization

An independent data monitoring committee is responsible for regularly monitoring patient safety, treatment efficacy, and overall study progress and for assessing whether early termination or protocol amendments are necessary.

## Discussion

In our previous pilot clinical trial investigating the safety and efficacy of remote ischemic conditioning for SAP, 19 patients with AIS onset within 48 h were randomly assigned to the RIC group (six consecutive days, twice daily), whereas 22 patients were assigned to the control group. The results of this study showed that RIC was safe among patients with AIS. Although the difference was not statistically significant in either univariate or multivariate analyses, the incidence of SAP was lower in the RIC group (10.5%) than in the control group (27.3%) [15]. The lack of statistical significance may be attributed to the small sample size, limiting the power to detect differences between groups, and recent evidence suggests that the peak of apoptosis and immune suppression following stroke may occur within the first 24 h [16], indicating that earlier prevention might be more beneficial among these patients. Therefore, we aimed to conduct a fully powered clinical trial with adjusted inclusion and exclusion criteria to investigate the efficacy of RIC in preventing SAP in AIS patients within 24 h of symptom onset following randomization. We hypothesize that 7 days of RIC will reduce the relative risk of SAP by 30%.

We implemented several adjustments to address the limitations identified in the previous study [15]. The inclusion criteria were refined to ensure that the treatment window was within 24 h of stroke onset, as this period is critical for effectively reducing apoptosis and modulating immune responses. Since the sample size calculation was based on previous studies [14], clinical expertise, and prior surveys on SAP incidence at Xuanwu Hospital of Capital Medical University and our participant selection criteria are relatively broad, we anticipate few practical challenges in recruiting a sufficient number of patients. An important advantage of our approach is that the results of our trial can be generalized to a wider population of stroke patients. Additionally, at the end of the study, we used a method from previous research [17] and contacted the participants, asking them about their knowledge of group allocation by answering the questions in Table 1. This upcoming trial is anticipated to conclude in the second quarter of 2025.

Owing to the low cost and ease of use of RIC devices, this intervention has strong potential for widespread adoption by patients and healthcare providers. If successful, this study could demonstrate that RIC significantly reduces the incidence of SAP, promotes recovery, and ultimately improves the quality of life for a large population of stroke patients.

## Trial status

The participant recruitment for this trial commenced on January 22, 2024, and is anticipated to conclude by the end of December 2024. This protocol is the third version, version date: July 19, 2023.

## Summary and conclusions

RIC is a convenient and noninvasive intervention, and the RICA-2 trial aims to investigate whether RIC treatment can effectively reduce stroke-associated pneumonia while also identifying any potential safety concerns.

## Supplementary Information

Additional file 1. SPIRIT checklist
